# Continuous Melt Granulation for Taste-Masking of Ibuprofen

**DOI:** 10.3390/pharmaceutics13060863

**Published:** 2021-06-11

**Authors:** Seth P. Forster, David B. Lebo

**Affiliations:** 1 Pharmaceutical Commercialization Technology, MMD, Merck & Co., Inc., Kenilworth, NJ 07033, USA; 2 Department of Pharmaceutical Sciences, School of Pharmacy, Temple University, Philadelphia, PA 19140, USA; david.lebo@temple.edu

**Keywords:** continuous processing, melt granulation, taste-masking, twin-screw granulation

## Abstract

Taste-masking of drugs, particularly to produce formulations for pediatric patients, can be challenging and require complex manufacturing approaches. The objective of this study was to produce taste-masked ibuprofen granules using a novel process, twin-screw melt granulation (TSMG). TSMG is an emerging, high-productivity, continuous process. Granules of ibuprofen embedded in a lipid matrix were produced across a range of process conditions, resulting in a range of output granule particle sizes. The ibuprofen appeared to be miscible with the lipid binder though it recrystallized after processing. The ibuprofen melt granules were tested in simulated saliva using a novel, small-volume dissolution technique with continuous acquisition of the ibuprofen concentration. The ibuprofen release from the granules was slower than the neat API and physical blend, beyond the expected residence time of the granules in the mouth. The ibuprofen release was inversely related to the granule size. A Noyes–Whitney dissolution model was used and the resulting dissolution rate constants correlated well with the granule size.

## 1. Introduction: The Need for Taste-Masked Formulations

Patient compliance is a profound public health challenge. One review estimated that patients in developing countries are non-compliant an average of 50% of the time for a chronic treatment [1]. In many cases, non-compliance leads to inadequate efficacy or disease mitigation and, particularly for infectious diseases, leads to resistance that is detrimental to the patient and public health [2]. While many social and economic incentives can help improve compliance, the development of ‘patient-centric’ dosage forms to improve the patient experience may also provide significant improvement in compliance and maintain a positive patient sentiment toward the product. A pleasant organoleptic formulation enabled by taste-masking of bitter drugs helps improve the patient experience, particularly for pediatric and elderly patients and any patient who has an aversion to or difficulty swallowing tablets [3]. Prior to the early 2000s, when legislative and regulatory changes in the United States and the European Union were put in place, most drug products were not evaluated for safety in children and not formulated for them. Now an age-appropriate formulation is required if the drug is intended to be dosed to children, which generally requires a palatability assessment [4,5].

### 1.1. Taste-Masking Formulation Approaches

Many taste-masking formulation technologies are available, as reviewed elsewhere [6,7,8,9]. Taste-masking approaches are briefly described below.

- Cover the flavor: Provided the API’s flavor is not too unpleasant and does not cause local irritation, the addition of flavors or sweeteners to the dosage form can be enough to improve the taste of the drug;
- Prevent dissolution by chemical means;
  ○ Drug solubility is insufficient in saliva;
  ○ Modification of the molecule to produce a prodrug that is not soluble in saliva;
  ○ Selection of a salt that is not soluble in saliva;
  ○ Complexation with excipients that prevent dissolution of the drug in saliva, e.g., cyclodextrins, ion-exchange resins, ionic polymers, often taking advantage of pH-dependent solubility;
- Prevent dissolution by forming a physical barrier to dissolution;
  ○ Matrix encapsulation where the drug is embedded in a lipid or polymer, typically a small quantity of drug is on the surface so a certain amount of drug dissolution in saliva must be tolerable;
  ○ Membrane encapsulation where the drug is completely isolated by a layer of lipid or polymer to prevent dissolution in saliva;
- Combinations of these techniques: for example, a membrane around a matrix particle with a flavor and sweetener system in an orally disintegrating tablet (ODT).

**Figure 1:**
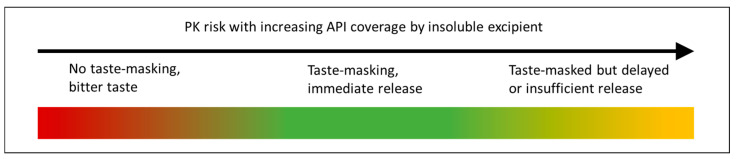
Schematic of balance between effective taste-masking and pharmacokinetic (PK) risk for dissolution barrier approaches in development of an immediate release (IR) product [10].

When developing a taste-masked dosage form, a bioequivalence (BE) strategy is typically preferred unless an improvement in onset time or efficacy is expected due to buccal or sublingual absorption or rapid disintegration. A BE approach requires an understanding of the trade-off between slowing the dissolution of the API in saliva and allowing for enough release in the gastrointestinal tract, as shown in Figure 1. Typically, it is necessary to avoid very slow release of the API to prevent reduced absorption and efficacy, but this may be acceptable for some drug products or where sustained release and taste-masking are both required.

### 1.2. Taste-Masking Assessment

Even with extensive research in taste-masking, the effectiveness of any given option is hard to predict. Because APIs are novel chemical structures, the taste receptor engagement is difficult to predict prior to formulation. Even if the taste of a compound were predictable, the safety and efficacy of the molecule are prioritized over taste in development. The issue of taste is left to the formulator to address. Furthermore, clinical evaluation in healthy patients is not always sufficient since many diseases can alter the perception of taste. For pediatric formulation development, adult patients can be sufficient to screen formulations for PK, but children have very different preferences for taste and dosage forms, generally more averse to bitter flavors and less readily able to swallow tablets [5,11].

With a specific API, preclinical in vitro or in vivo models of the taste of the drug are not usually predictive. Clinical evaluation has been recommended to assess the drug concentration threshold of acceptability [12]. However, direct clinical assessment is expensive and for a new chemical entity, involves ethical and toxicology assessment. Analytical methods are much easier to use, though more research is required to improve their ability to predict clinical taste-masking performance. Electronic tongue diagnostic techniques can help assess the intensity of the API’s bitterness or comparing matrix tastes and has been correlated with human taste panel results [13]. However, the equipment is complex and not readily adapted to continuous monitoring or short, biorelevant sampling times. Models for buccal dissolution and conventional USP I or II apparatus dissolution in biorelevant media can be used to screen formulations and to guide development.

Several approaches have been proposed for in vitro testing of taste-masking. A typical strategy for taste-masking is to select a target threshold of API release and an estimated time in the mouth. These estimates tend to be arbitrary but can still provide a useful comparison across formulation or process parameters to guide clinical testing. For example, a joint International Pharmaceutical Federation (FIP)/American Association of Pharmaceutical Scientists (AAPS) guideline proposed that taste-masking was successful if not more than 10% of the API released within five minutes of dissolution [14]. Clinical success with formulations that pass this metric depends on the bitterness of the API and does not consider the impact of flavors or sweeteners.

Quality control methods for generic drugs described by the United States Pharmacopeia (USP) tend to use large volumes of dissolution media, typically 250 mL to 1 L, stirred gently. These methods are neither expected to discriminate subtle changes in API release nor appropriate for characterization of taste-masking risk since the volume of saliva in the mouth is quite low at any time, less than 1 mL [15]. Therefore, the local concentration is likely to be higher. Since saliva flows into and out of the mouth most of the time, ranging from 0.25 mL/min unstimulated to 2 mL/min in the presence of food [16], the API is likely reduced and swallowed.

Pein et al. reviewed taste-masking dissolution studies [12]. Most of the dissolution techniques described reasonable sampling times ranging from fifteen seconds to three minutes to the first sample, though some sampling times were even shorter or had durations as long as ten minutes. The actual time in the mouth is longer than several seconds but likely shorter than a minute, even for patients with difficulty swallowing tablets. Beck et al. trained children with demonstrated tablet anxiety to swallow tablets within thirty seconds [17].

Many of the dissolution studies reviewed use discrete measurements that are then tested off-line. An example of a single-point test is described by Hamashita et al. [18] where granules of ibuprofen were coated to varying levels, then placed in 20 mL of media for ten seconds and removed. This approach masks the large differences in release profiles that could occur across that time and could impact clinical taste-masking and highlight the benefit of continuous measurement in the first few minutes of release. Some of the taste-masking dissolution studies in the review had threshold targets selected based on human taste studies, but these also tend to depend on the study subjects and matrix effects and can vary widely. For example, different thresholds have been reported for acetaminophen, 0.35 mg/mL [19] and 1.08 mg/mL [20].

### 1.3. Continuous Twin-Screw Melt Granulation for Taste-Masking

Among the drug product processing techniques, melt granulation is well suited to taste-mask APIs by matrix encapsulation. As the molten binder is heated, it tends to preferentially cover the outer surface with reduced exposed surface API. This typically results in reduced drug release at the same drug loading level [21,22]. Lipids such as glyceryl monostearate [23], glyceryl distearate [24], glyceryl dibehenate [25], hydrophilic matrix-forming polymers such as hydroxypropyl cellulose [26], and the amino-methacrylate copolymer Eudragit^®^ E [23,27] are well suited for this matrix encapsulation by melt granulation.

Melt granulation can leverage many of the process trains used for wet granulation, including high shear and fluid-bed granulators. Compared with these other process techniques, twin-screw melt granulation (TSMG) is of interest since it is a continuous process. Continuous equipment has smaller process footprint, improved batch size flexibility by allowing scaling by time, and improved in-process monitoring and control. Continuous processing puts less material at risk and reduces downtime. Continuous processes are expected to result in improved quality and lower cost of production [28,29,30]. Specifically, TSMG has improved control of rate of material addition, heat, and shear during granulation compared with batch melt granulation [31]. It can be designed to greatly reduce material residence time at temperature, enabling higher process temperatures without causing API or excipient degradation [32]. The modular screw and barrel design, common among twin-screw extruders, allows for improved control of granule density and particle size. For more information, refer to recent reviews of continuous melt granulation [10,31] and process studies [33,34,35,36]. TSMG has similar process considerations to twin-screw wet granulation, which is comparatively more developed in the literature [37,38,39,40,41,42].

The goal of this work is to demonstrate the TSMG process with a model API, ibuprofen, and observe the impact on granule properties across the process space studied. These granules were tested with a simple simulated saliva dissolution method and compared to reference pediatric ibuprofen formulations.

## 2. Materials and Methods

### 2.1. Materials

Glyceryl distearate (Precirol ATO 5^®^, Gattefossé) was used as the lipid binder and taste-masking agent. It has a relatively low melting range of 50–60 °C, which reduces the required process temperature and therefore the risk of chemical or physical instability. Precirol has a mean particle size of 35–65 µm.

Ibuprofen (USP grade, Spectrum Chemical, New Brunswick, NJ, USA) is a common non-steroidal anti-inflammatory drug (NSAID). It has a low melting point of 75–77 °C [43].

Junior Strength Advil^®^ Chewables and Children’s Motrin^®^ Chewable Tablets were purchased over the counter. Both were 100 mg strength scored tablets that were split in half for dissolution testing in order to match the strength of granules tested.

Simulated saliva was prepared as described by Hughes and Gehris [44], in short, a phophate buffer with calcium chloride adjusted to pH 6.2 to mimic the ionic strength and pH of saliva. Dissolution media components were purchased USP grade from Fisher. Deionized water was used to prepare the media.

### 2.2. Formulation Screening

In order to assess the impact of drug loading on the dissolution, melt granules of several drug loadings of ibuprofen were prepared using a small twin-screw extruder. A pre-blend was prepared by tumble blending (Turbula, 5 min at 46 rpm). The pre-blend was fed with a vibratory feeder (VIBRI, Sympatec, Inc., Pennington, NJ, USA) into a custom corotating twin-screw extruder with 7.5 mm diameter screws and 150 mm length barrel, that is, 20:1 L/D (screw length to diameter ratio). The screw profile was predominantly conveying with a single mixing zone of three forward mixing paddles of 1/3 D each with 90° offset. The extruder was run without a die. The feed zone was water cooled and kept near room temperature. Three controlled heating zones were set to 45–60 °C. The output granules cooled at ambient conditions. After storage, the granules were sieved through a #20 mesh/0.85 mm screen to remove large particles prior to testing.

### 2.3. Pilot-Scale Melt Granulation

Pilot-scale melt granulation was performed using a Thermo Prism 16 mm twin-screw extruder in 40:1 L/D configuration. Pre-blends were prepared at 1 kg batch size using a tote blender (L.B. Bohle 4 L tote, 10 min at 25 rpm). The blend was fed at a controlled rate into the hopper feed throat using a loss-in-weight (LIW) feeder (Coperion K-Tron KT20 with coarse concave screws). The extrusion screw profile had limited mixing with three 4 mm (L = D/4) forward kneading block elements at 30° offset located at Zone 5. The rest of the screw was conveying, with long pitch screw at the feed section (Zone 2), standard pitch for most of the profile, and discharge screws at the exit (Zone 10). The barrel zones around the mixing section were heated to target temperatures around the melting point of the binder (50–60 °C) and the downstream zones were cooled. See Table 1 for process design and zone temperatures. The process was run at varying screw speeds with open screw discharge to avoid consolidation in the die. The heating zone temperatures, screw speed, and blend throughput were adjusted (Table 2). A design of experiments (DOE) approach was used with the center point condition repeated to estimate reproducibility. Motor torque and power readings were recorded from the Thermo Prism 16 mm extruder. The outlet temperature was also recorded from the extruder. Granule samples of 25–50 g were collected after 5–10 min of processing when the granules were qualitatively similar and motor torque and power were stable. Typical mean residence times for this equipment were less than one minute [45], so this corresponds with at least five mean residence times.

### 2.4. Granule Characterization

Particle size distribution of the granules was measured in triplicate using the Sympatec QIPCPIC image analysis and laser diffraction system with a RODOS disperser feed using a VIBRI vibratory feeder. The dispersion pressure was 2.0 bar. The M7 lens with a 10–3410 µm measurement range was used. Samples were manually screened through a 2.0 mm sieve prior to testing.

Scanning electron microscopy (SEM) was performed with the FEI Quanta 250 in environmental (ESEM) mode.

### 2.5. Physical Form of Ibuprofen in Melt Granules

Since ibuprofen melts at a low temperature and is known to form eutectics or solid solutions with polymers [46,47], X-ray powder diffraction (XRPD) was used to assess the risk of ibuprofen physical instability or phase changes due to the granulation process. XRPD was performed at ambient conditions with a PANalytical X’Pert powder X-ray diffractometer in a transmission configuration. The samples were scanned from 2 to 40° 2θ at a rate of 2.5°/min. Data acquisition and analysis were performed with Data Collector and PANviewer software.

The physical form of ibuprofen, particularly to assess whether two melting points are observed and the melting point depression of ibuprofen in the presence of Precirol, was evaluated using differential scanning calorimetry (DSC, Q2000, TA Instruments, New Castle, DE, USA). The samples were placed in non-hermetically sealed, closed pans, equilibrated to 20 °C, then heated a single-pass 5 °C/min to 120 °C. Data analysis was performed with TA Universal Analysis software.

### 2.6. Small-Volume Dissolution Method

As a tool for formulation screening, a small-volume, aggressively stirred dissolution method was developed. A volume of 20 mL was selected as a practical compromise with the biologically relevant volume in order to facilitate constant monitoring of drug concentration. This methodology is intended to compare the variations in drug release in a consistent way. It is not necessarily predictive of taste-masking functionality, in fact, the high degree of agitation is likely to result in significantly more drug release than when dosed in the mouth. Therefore, if the melt granules can reduce the release for thirty seconds to a few minutes, then they are expected to exhibit better taste-masking in vivo.

**Figure 2:**
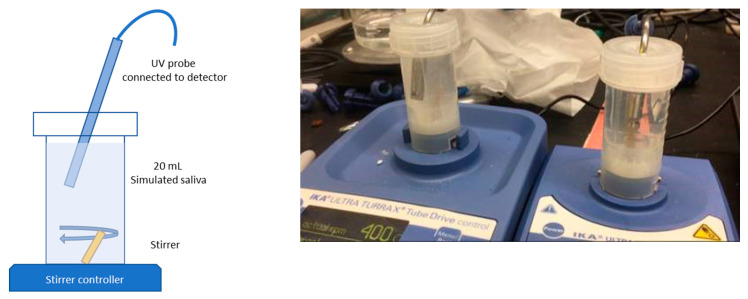
Dissolution set up with IKA Ultra-Turrax^®^ mixers.

Refer to Figure 2 for the dissolution setup. An IKA Ultra-Turrax^®^ mixer with a 25 mL tube containing a rod impeller was used at 4000 rpm. A simplified simulated saliva fluid was used, described in the Materials section [44], that had been degassed by preheating to 45 °C. Upon aliquoting the media, it cooled to 37 ± 2 °C before commencing the dissolution test. The mixing chamber temperature was not controlled, though the time between aliquoting the liquid and completion of the test was less than 10 min. Drug concentration was monitored continuously. An immersion probe with fiber optic connection to a Pion Rainbow^®^ Dynamic Dissolution Monitor ultraviolet-visible light spectrophotometer, with an acquisition range of 200–720 nm, controlled using the Pion AuPro software. The path length on the probe was optimized at 5 mm for ibuprofen. Sufficient formulation was used for a theoretical strength of 50 mg of ibuprofen. Drug concentration was determined using a calibration curve based on the 2nd derivative of the signal from 264 to 300 nm. All dissolution tests were performed in triplicate.

The time and concentration target for ibuprofen for acceptable taste have not been directly determined. Hamashita et al. reported volunteers did not taste coated ibuprofen granules that released up to 1000 µg/mL after a 10 s rapid dissolution test [18]. For this study, a more stringent taste-masking target of not more than 5% label claim ibuprofen (5% × 50 mg/20 mL = 125 µg/mL) release at one minute was used. This is intended as a working target to screen formulations, which could be validated by a human taste study.

## 3. Results

### 3.1. Protoype Process to Screen Drug Loading

**Figure 3:**
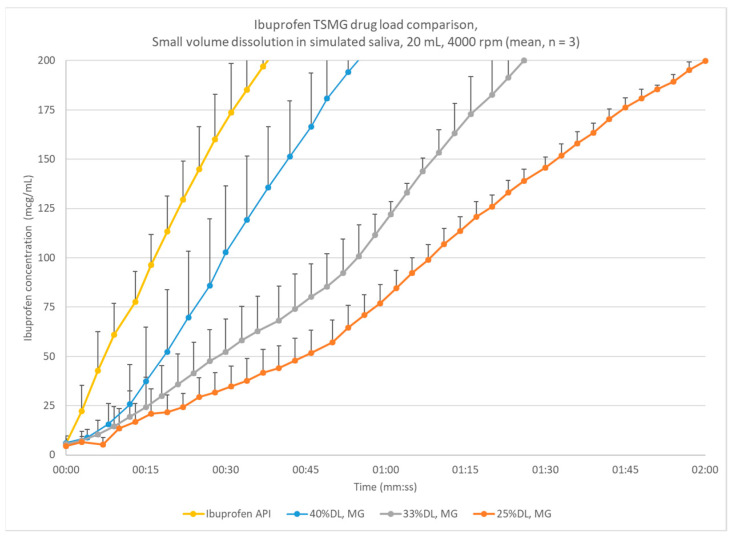
Small-volume dissolution comparison of prototype ibuprofen melt granulations across drug loadings, produced at the 7.5 mm scale; error bars indicate one standard deviation.

Granules were produced at 25%, 33%, and 40% DL ibuprofen. They were qualitatively similar and had similar particle size distributions. Small-volume dissolution indicated that the 33% and 40% DL test granulations had slowed time to the target threshold to 15–60 s (Figure 3). These concentrations were selected for scale-up.

**Figure 4:**
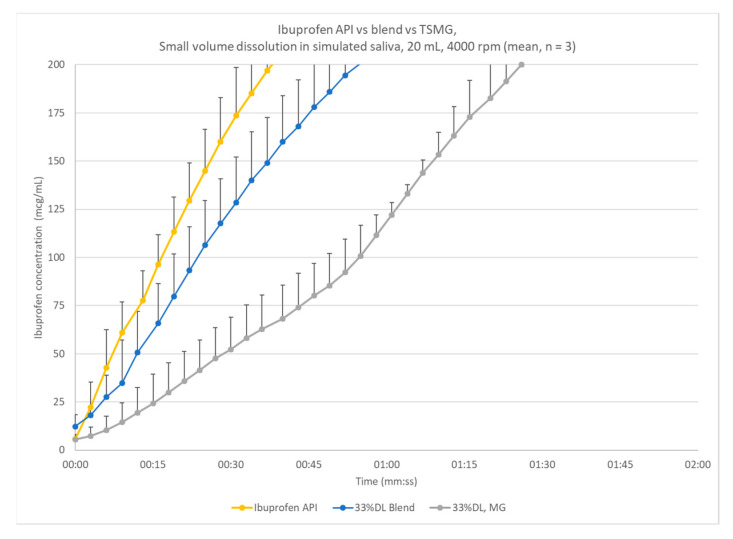
Small-volume dissolution comparison of ibuprofen to 33% DL blend and melt granulation produced at the 7.5 mm scale; error bars indicate one standard deviation.

To assess the impact of granulation on rate of dissolution, 33% DL melt granules and blend were compared with the neat ibuprofen and a physical blend. Ibuprofen releases rapidly, though slowing as it approaches its equilibrium solubility at pH 6.2 (Figure 4). The blend with Precirol reduces the release only slightly while the melt granules produced at the prototype scale are approximately 125 mcg/mL at one minute.

### 3.2. Pilot-Scale Process with 33% DL and 40% DL Ibuprofen

For both the 33% DL and 40% DL formulations, the process responses of motor power and torque were directly related to the screw speed, consistent with typical twin-screw extrusion processes. Of the process parameters, only screw speed was statistically significant in predicting the power or torque. Both parameters were relatively low compared to polymer or amorphous solid dispersion applications since the barrel was not fully filled and the system is not highly viscous. Torque was < 5 N-m and power < 50 W across all the conditions.

### 3.3. Granule Particle Size and Morphology

Granule characterization was focused on the particle size distribution as this was directly related to the dissolution of the API as well as the granule morphology. The particle size distributions of all the batches are described in Table 3 and Table 4. Many of the batches are bimodal with a fine mode around the starting Precirol^®^ particle size of d50 = 35–65 μm and a coarse mode in the 300–1100 µm range. As the extent of granulation increases, more of the batch is in the coarse fraction and the average of that mode increases. Though it depends on the matrix and the test population, a reference threshold for ODTs with core granules of particle size for feeling the roughness in the mouth is approximately 200–300 μm [48]. The process output could be sieved to remove particles above this threshold to avoid grittiness, with a corresponding loss in yield.

**Figure 5:**
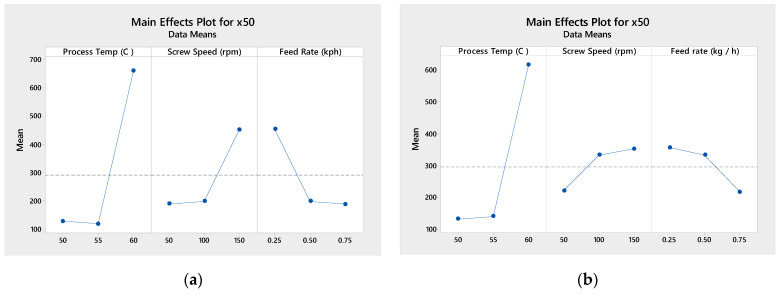
Main effects plots for mean particle size (×50) for 33% DL (**a**) and 40% DL (**b**) ibuprofen TSMG, includes results across screw speeds.

Main effect plots (Figure 5) shows the relative impact of the different process variables on the output granule mean diameter. The process temperature has the largest impact on mean granule size, particularly from 55 to 60 °C. The granule size increases with increasing screw speed and reduced feed rate at both drug loadings, though with a more pronounced effect for the 33% DL.

**Figure 6:**
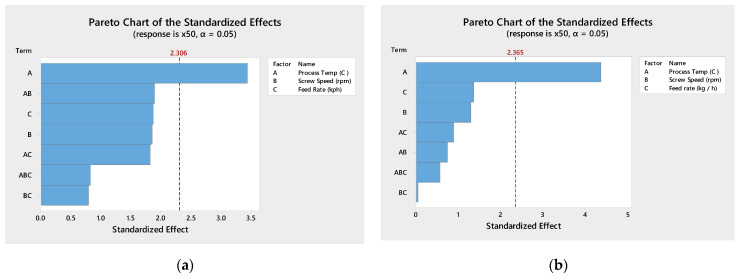
Pareto chart for 33% DL (**a**) and 40% DL (**b**) ibuprofen TSMG.

**Figure 7:**
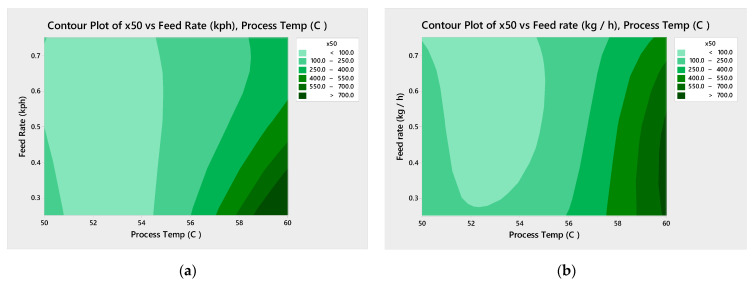
Contour plots for mean particle size (×50) for 33% DL (**a**) and 40% DL (**b**) ibuprofen TSMG, includes results across screw speeds.

Figure 6 shows Pareto analysis for both drug loading studied shows that the process temperature is statistically significant and the most impactful factor in predicting the mean particle size (Figure 6). The screw speed is also statistically significant for the 33% DL formulation. The contour plots (Figure 7) confirm that higher process temperatures result in larger granules. The mean particle size (×50) is used in this analysis, though the tenth (×10) and ninetieth (×90) percentile particle sizes follow similar trends.

**Figure 8:**
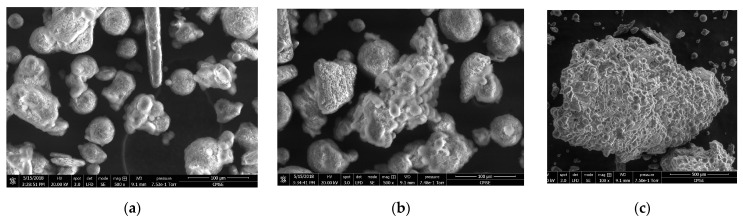
The 33% DL ibuprofen melt granules showing range of extent of granulation, left to right. Process conditions: (**a**) 50 °C, 150 rpm, 0.25 kph at 500×; (**b**) 55 °C, 100 rpm, 0.5 kph, center point at 500×; (**c**) 60 °C, 50 rpm, 0.25 kph at 100×.

Scanning electron micrographs (SEMs) of the granules show a similar trend in particle size for both drug loadings. Figure 8 shows the 33% DL granules with varying extent of granulation, from predominantly primary particles to large granules of several millimeters. The particle morphology confirms the granule growth behavior with increasing time at temperature: initially ungranulated lipid is fine, spherical, smooth particles and ibuprofen is larger rod or cubic particles; as the lipid melts, it covers the ibuprofen particles and, with more melting, the granules coalesce into larger granules. The granule morphology is similar qualitatively between the two drug loadings.

### 3.4. Physical Characterization

**Figure 9:**
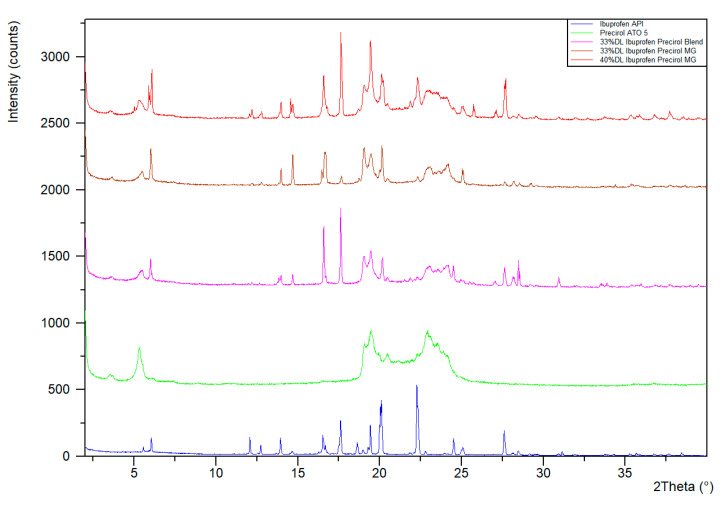
XRPD of ibuprofen granulations.

Figure 9 shows that both 33% DL and 40% DL ibuprofen granules have XRPD signals consistent with the neat crystalline ibuprofen API. Glyceryl distearate is a crystalline lipid with diffraction in the range of 18–25° 2 θ. Ibuprofen has several strong diffraction peaks across the range, e.g., 17.5, 20, 27.5° 2 θ.

**Figure 10:**
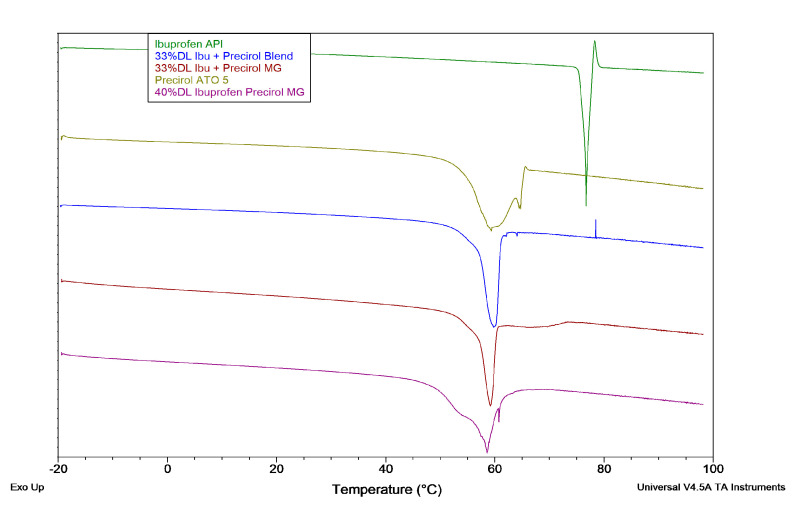
DSC of ibuprofen granulations.

Single-pass DSC (Figure 10) results show several melting peaks for Precirol, consistent with observations of multiple crystalline phases reported elsewhere [49]. Neat ibuprofen shows a clear melting point at the expected temperature. The 33%DL blend and granulation have melting points around the temperature for neat Precirol but not for ibuprofen. Together, these results suggest that the ibuprofen is dissolving into the Precirol during heating and recrystallizing after cooling and storage.

### 3.5. Small-Volume Dissolution

Small-volume dissolution with continuous concentration measurement was used to compare prototype-scale formulations and select the drug loadings for futher study. Figure 7 shows the impact of drug loading for the ibuprofen prototype formulations. As mentioned previously, 5% LC or 125 mcg/mL of ibuprofen was used as a target ibuprofen concentration threshold. The 25% DL melt granules release much more slowly than 33% DL, and the 40% DL melt granules release more quickly, though still slowed compared with the neat ibuprofen release. These tests used granules from the prototype-scale 7.5 mm extruder, so the particle size may not be representative of what is observed at pilot or commercial scale. Since a higher drug loading corresponds with a lower overall formulation mass and the impact of scale-up and process parameters, the 33% DL and 40% DL formulations were scaled up.

**Figure 11:**
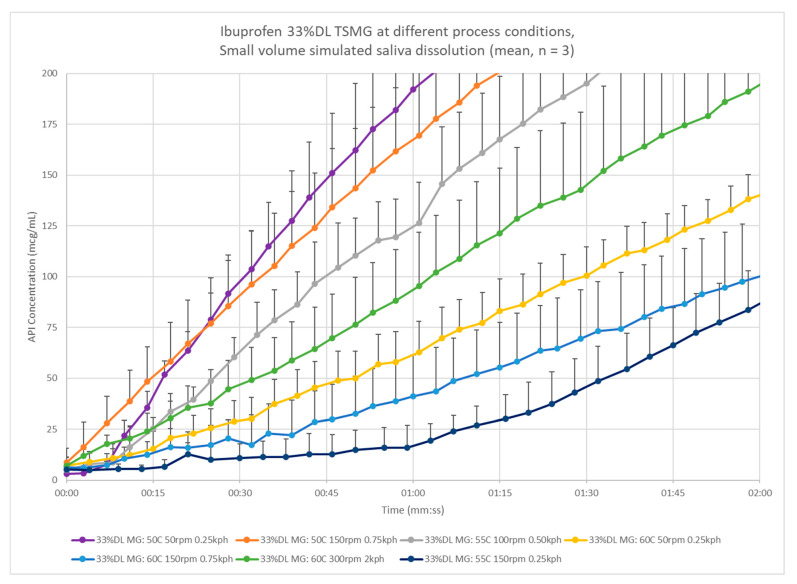
Small-volume dissolution comparison of 33% DL ibuprofen melt granulations across pilot-scale process conditions.

The 33% DL and 40% DL ibuprofen melt granulation was scaled up to pilot scale, varying the process temperature, screw speed, and pre-blend feed rate. The resulting granules varied in size distribution, trending towards larger granules at higher temperatures and longer residence times. The dissolution rate of the ibuprofen from the granules is extended as well with larger granule particle size as shown in Figure 11. The dissolution release rate increases with larger mean particle diameters. The time to 5% LC release ranges from thirty seconds up to two minutes and thirty seconds. The center point condition (55 °C, 100 rpm, 0.5 kph) reaches the threshold concentration at about one minute.

**Figure 12:**
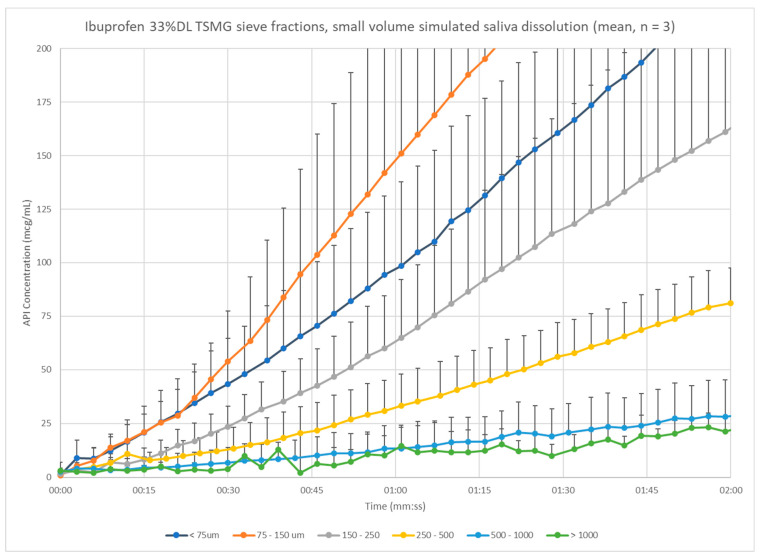
Small-volume dissolution comparison across sieve fractions of 33% DL ibuprofen granules.

To confirm the impact of particle size on the ibuprofen dissolution, the pilot process center point batch from the 33% DL ibuprofen melt granules was sieved and the small-volume dissolution tested. The sieve fraction particle sizes cover a wider range of mean particle sizes than the process batches previously studied, and so have a wider range of times to the threshold target of 5% LC, from fifty seconds to more than ten minutes (Figure 12). The coarser granules release more slowly than the finer granules, except for the finest fraction of less than 75 µm. The cause of the slower than expected release of the finest particles is not clear, though it may be due to poor wetting of the powder in the media.

**Figure 13:**
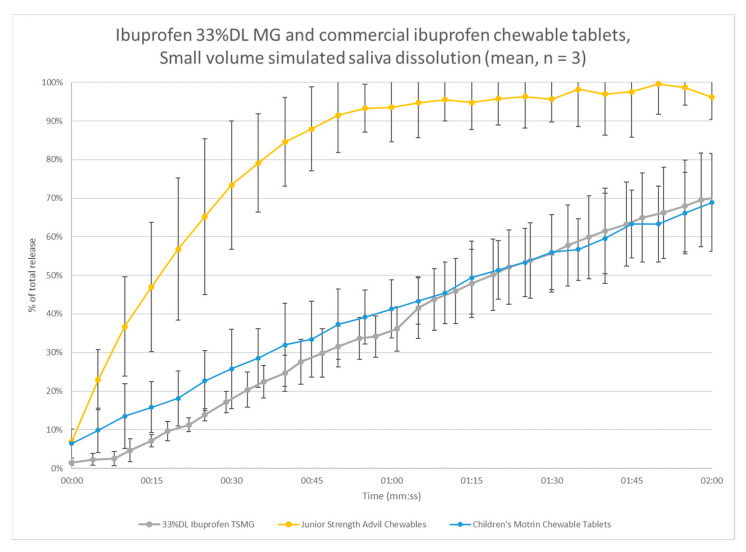
Small-volume dissolution comparison of ibuprofen melt granules and commercial chewable tablets.

Two commercial ibuprofen products, Junior Strength Advil^®^ Chewables and Children’s Motrin^®^ Chewable Tablets, were tested a compared with the 33% DL melt granules from the pilot-scale center point batch (Figure 13). These tablets were split along the score to test at 50 mg strength. These data are reported as percent of total release to account for differences in the formulations. The chewable tablets may have differences in potency or different excipients that alter the pH of the dissolution media or the UV absorption. The results show similar rate of release for the melt granules compared with the Motrin^®^ product but much slower than the Advil^®^ product. Bearing in mind that the reference products were tested as tablets, with a much lower exposed surface area, they would be expected to release much slower than the granules. This result supports similar or better taste-masking of ibuprofen compared with these products.

## 4. Analysis

### 4.1. Continuous Melt Granulation Mechanism

Melt granule growth has previously been described as proceeding by ‘distribution’ where the binder melts to coat the other particles and then impacts result in coalescence or by ‘immersion’ where the surface of the binder particle softens so the other particles adhere to the outside of the binder particle. The immersion mechanism is promoted by a smaller binder particle size relative to the other components and a lower binder melt viscosity. The distribution mechanism is promoted by a larger particle size and higher binder melt viscosity [50]. These mechanisms are growth regimes for wet granulation as well [51].

The ibuprofen/Precirol melt granulation characterization results are consistent with the distribution mechanism of melt granulation. The Precirol has a small binder particle size relative to the API particles and a low binder viscosity upon melting.

### 4.2. Granulation Process Analysis

Beyond the primary process parameters, alternative-scale independent factors have been proposed to allow for comparison of screw designs and to aid scale-up for amorphous solid dispersion processing with twin-screw extrusion [52], emphasizing thermal energy, related to temperature in the processing zones, mechanical energy imparted by the screws, and the amount of material in the process zone as mass throughput or fill level in the extruder. For twin-screw wet granulation [53], thermal energy input is generally low and therefore not considered though mechanical energy input and fill were critical. For twin-screw melt granulation, a recent publication suggested that, other than temperature, closely monitoring two parameters, fill level and specific mechanical energy, were critical when comparing across screw and equipment designs [34].

Twin-screw granulation studies from the literature report that granule attributes can be predicted from the extruder channel fill level [41,54,55]. However, the actual extruder channel free volumes and material densities in situ is difficult to measure. The specific throughput (ST), that is, the ratio of the feed rate and the screw speed, can be used as a simple estimate of fill level, and has been found to correlate well to degree of fill as well as granule quality attributes [56].

Granule properties have also been related to the specific mechanical energy (SME) imparted by the screws [38,57]: SME = 2π τ ω/ṁ, in J/g, where τ = torque (N-m), ω = screw speed (rpm), and ṁ = mass flow rate (g/min).

**Figure 14:**
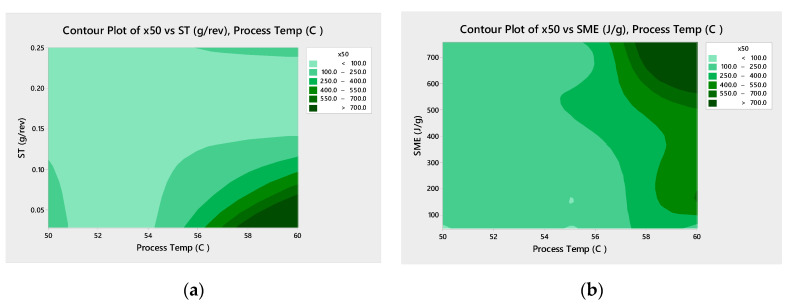
Contour plots of ×50 vs. ST (**a**) and SME (**b**) with process temperature, 33% DL ibuprofen TSMG.

**Figure 15:**
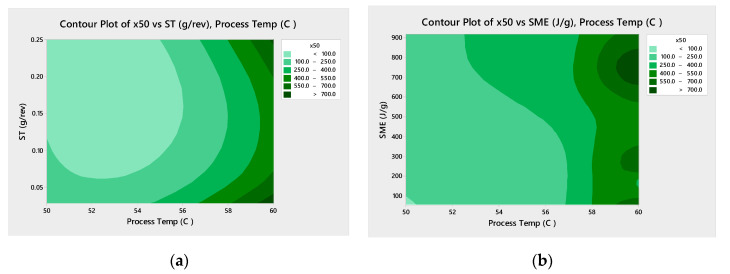
Contour plots of ×50 vs. ST (**a**) and SME (**b**) with process temperature, 40% DL ibuprofen TSMG.

The ST and SME for the process conditions used for 33% DL and 40% DL ibuprofen granule batches were calculated. Contour plots below (Figure 14 and Figure 15) plot the mean granule size (×50) against temperature, ST, and SME. For 33% DL, the low ST at high temperature has the largest granules, as expected since this means less material in the extruder with each revolution and more heat input per unit mass. For 40% DL, more granulation is also observed at the higher ST and 60 °C, perhaps as the barrel is filled to the point of compacting the material.

### 4.3. Dissolution Model for Ibuprofen Melt Granulation

**Figure 16:**
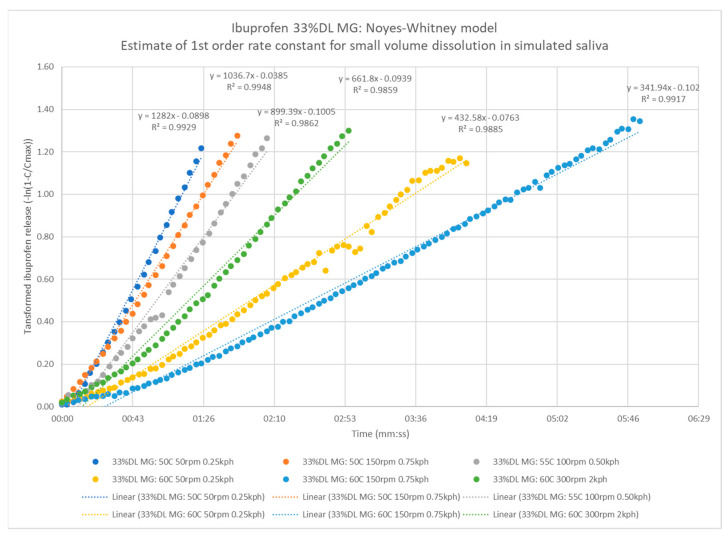
Example Noyes–Whitney model fit for ibuprofen 33% DL melt granules.

The results from the process study batches and sieve fractions indicate a strong impact of granule particle size on dissolution. The Noyes–Whitney equation (Equation (1)) is often used to correlate the dissolution rate with formulation attributes. An integrated solution allows for estimation of the dissolution rate constant (Equation (2)) [58]. This model is the same as a first order continuously stirred tank reactor (CSTR). Ibuprofen release data were transformed to plot and regression fit -ln(1-C/Cmax) vs. time (Figure 16). The slope of the regression is the dissolution rate constant k in 1/d, equivalent to KS_w_ shown in Equation (2). For all the samples, the correlation coefficient is very good, R^2^ > 0.95.

(1) $$\frac{dC}{dt}=\frac{DS}{Vh}\left(C_{s}-C\right)$$

(2) $$C\left(t\right)=C_{s}\left\{1-\exp\left(-\frac{D}{Vh}St\right)\right\}$$

where *C(t)* is the concentration of the API at a given time t,

*C* is the solubility concentration of the API,

*D* is the diffusion coefficient of the API in the media,

*S* is the exposed surface area of the API,

*V* is the volume of the solution,

*h* is the diffusion boundary layer thickness, and

**Figure 17:**
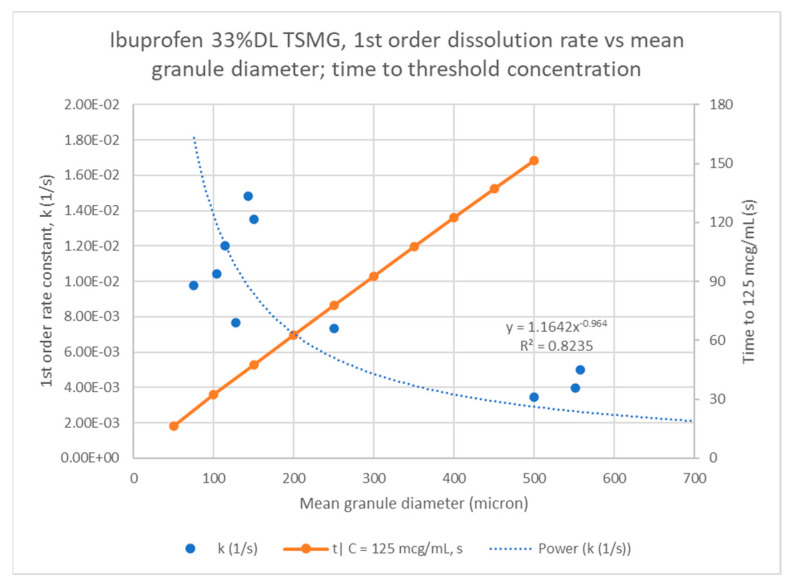
Noyes–Whitney rate constant correlation to mean particle size for ibuprofen 33% DL melt granules.

**Figure 18:**
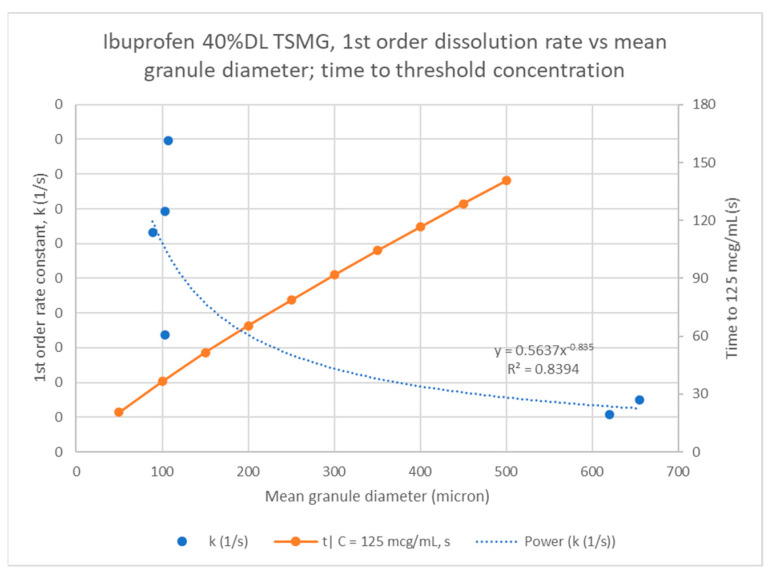
Noyes–Whitney rate constant correlation to mean particle size for ibuprofen 33% DL and 40% DL melt granules.

Figure 17 shows a power law fit of the rate constants to mean granule particle size has a good correlation (R^2^ = 0.8). This fits with the Noyes–Whitney model expectation since the API exposed surface area is likely to be related to the mean granule particle size. Using the regressed model, an estimate of the time to the threshold concentration for ibuprofen of 125 µg/mL or 5% LC is in the range of approximately one to three minutes across typical granule particle size range, 100–500 µm, which is consistent with what was observed empirically during dissolution. Figure 18 shows the same analysis for 40% DL ibuprofen melt granules.

The predicted time to the target ibuprofen concentration threshold (125 µg/mL) is similar for these two drug loadings across mean granule sizes. The model predicts that granules with mean diameters of approximately 200 µm or more will not reach the threshold within one minute for either drug loading. If two minutes of coverage are required, the 33% DL can accept slightly smaller granules of 400 µm mean diameter vs. 450 µm for 40% DL. These results demonstrate the balance of increasing drug loading, and thereby the final dosage form, against the granule size. The usable range of granule sizes is reduced as drug loading or taste-masking time increases. With a target granule size range, the granulation process may be optimized to improve the usable yield.

## 5. Conclusions

Continuous twin-screw melt granulation was demonstrated with ibuprofen using glyceryl distearate as a melt binder at the pilot scale. Granule particle size distribution and morphology were shown to be a strong function of the extrusion process parameters, particularly process temperature. The ibuprofen appears to be miscible with the lipid in the molten state though crystalline in the granules.

A simple, small-volume, aggressively stirred dissolution method using simulated saliva was developed and used to test the melt granules. From this dissolution test, the granules slowed ibuprofen release relative to the neat API and physical blend. Ibuprofen release was slower than reference commercial ibuprofen products, potentially indicating better taste-masking. Release rate was a function of granule particle size. The ibuprofen release profiles were fit using a Noyes–Whitney model and the resulting dissolution rate constant correlated well with particle size.

Future work is warranted to focus on the impact of other APIs, screw design, and scale-up, along the lines of similar work with twin-screw wet granulation [37,39,42]. Formulation of the granules into final dosage forms such as chewable or orally disintegrating tablets to assess the robustness of the granules to subsequent processing and impact on final performance would also be beneficial. Finally, improving the novel dissolution test by comparing in vitro results to taster panel data, either for this formulation or others, would help assess the applicability of the method and could be used to improve the predictive performance of the method.

## Figures and Tables

**Table 1:** Pilot-scale TSMG set up and zone temperatures.

Zone	1	2	3	4	5	6	7	8	9	10
Screw and barrel design	Vent	Feed	Convey	Convey	Convey to Mix	Convey	Convey	Convey	Convey	Convey to Discharge
Temperature set point (°C)	25	25	25	50–60	50–60	50–60	50–60	40	30	30

**Table 2:** Pilot-scale melt granulation process conditions.

	Temperature (°C)	Screw Speed (rpm)	Throughput (kg/h)
Low	50	50	0.25
Center	55	100	0.50
High	60	150	0.75

**Table 3:** Particle size distributions of 33% DL ibuprofen melt granules.

Process Temperature (°C)	Screw Speed (rpm)	Feed Rate (kg/h)	Average (µm)			Standard Deviation (µm)		
			×10	×50	×90	×10	×50	×90
50	50	0.25	55	143	1543	1.7	27	198
50	50	0.75	48	91	898	1.0	5	441
50	100	0.5	50	100	607	3.2	8	458
50	150	0.25	57	194	1147	2.1	68	320
50	150	0.75	53	114	1155	1.2	5	144
55	50	0.25	50	99	1152	1.0	2	317
55	50	0.75	48	93	1334	1.5	5	398
55	100	0.5	50	107	1571	1.0	7	364
55	100	0.5	51	104	1170	2.3	12	549
55	150	0.25	57	183	1071	2.0	22	178
55	150	0.75	52	126	1476	3.0	31	480
60	50	0.25	80	557	1359	8.2	217	502
60	50	0.75	58	159	982	1.2	13	143
60	100	0.5	92	486	959	11.7	66	89
60	150	0.25	566	1545	2331	95.2	191	70
60	150	0.75	106	551	1023	23.1	44	56

**Table 4:** Particle size distributions of 40% DL ibuprofen melt granules.

Process Temperature (°C)	Screw Speed (rpm)	Feed rate (kg/h)	Average (µm)			Standard Deviation (µm)		
			×10	×50	×90	×10	×50	×90
50	50	0.25	49	103	968	2.1	19	574
50	50	0.75	47	89	1209	1.0	4	612
50	100	0.5	54	165	1031	2.6	52	265
50	150	0.25	59	205	862	3.0	62	210
50	150	0.75	50	103	981	0.6	5	316
55	50	0.25	51	107	819	1.5	6	196
55	50	0.75	53	100	1306	5.3	8	250
55	100	0.5	51	107	1156	1.2	6	240
55	150	0.25	68	272	843	8.4	86	137
55	150	0.75	54	120	1005	1.0	4	274
60	50	0.25	88	655	1410	10.0	31	224
60	50	0.75	64	278	1154	3.1	78	354
60	100	0.5	90	730	1764	12.2	177	400
60	150	0.25	187	800	1319	58.4	80	245
60	150	0.75	96	620	1431	2.6	34	431

## Data Availability

The data presented in this study are available on request from the corresponding author.
